# A novel coumarin derivative treatment ameliorates neuroinflammation and cognitive functions in the ICV‐STZ‐induced rat model of Alzheimer’s disease

**DOI:** 10.1002/alz.090252

**Published:** 2025-01-03

**Authors:** PRAJJWAL SHARMA

**Affiliations:** ^1^ Central University of Punjab, Bathinda, Punjab India

## Abstract

**Background:**

Neuroinflammation has gained substantial attention for its involvement in the progression of Alzheimer’s disease (AD).

**Method:**

Recently, it was discovered that platelets, which make up 90% of the circulatory Aβ, encourage the development of AD. Therefore, we hypothesized to use a novel coumarin derivative PS21HKR, which is believed to possess anti‐platelet and anti‐inflammatory properties for the treatment of AD. We investigated the effects of PS21HKR on STZ induced cognitive decline and neuroinflammation. Intra‐cerebroventricular streptozotocin (ICV‐STZ) (3mg/Kg) induced Wistar rat models of AD were used in the study. Rats were divided into 3 groups including sham, STZ and PS21HKR group.

**Result:**

Behavioural studies revealed that PS21HKR mitigates the cognitive deficit associated with STZ. Anti‐platelet effect of PS21HKR was confirmed by tail vein bleeding assay. Further, *in‐vitro* studies were performed on BV2 cells including the MTT assay and determination of expression of inflammatory markers. The results suggested that PS21HKR potentially regulated the expression of pro‐ and anti‐inflammatory cytokines.

**Conclusion:**

Therefore, findings of this study indicate that PS21HKR has a potent neuroprotective effect by virtue of its anti‐platelet and anti‐inflammatory activity.

